# Improving Blood Pressure Control Using Digital Communication Methods in Serbia

**DOI:** 10.3390/diagnostics12040914

**Published:** 2022-04-06

**Authors:** Nebojsa Tasic, Danijela Tasic, Zorana Kovacevic, Marko Filipovic, Milan Arsic, Sladjana Bozovic-Ogarevic, Biljana Despotovic, Milovan Bojic, Zlatko Maksimovic, Nebojsa Zdravkovic, Sara Mijailovic, Vladimir Zivkovic, Tamara Nikolic Turnic, Vladimir Jakovljevic

**Affiliations:** 1 Institute for Cardiovascular Diseases Dedinje, 5 Heroja Milana Tepica Street, 11000 Belgrade, Serbia; nebtasa@yahoo.com (N.T.); dtasic74@yahoo.com (D.T.); zoca005@gmail.com (Z.K.); filipovicmarko76@gmail.com (M.F.); arsicdr@gmail.com (M.A.); sladjanaogarevic@gmail.com (S.B.-O.); dudic.z@sbb.rs (B.D.); dedinje@ikvbd.com (M.B.); 2 General Health Center “Sveti Vračevi”, 53 Srpske Vojske Street, 76300 Bijeljina, Bosnia and Herzegovina; zlatko.maksimovic@gmail.com; 3 Department of Medical Statistics and Informatics, Faculty of Medical Sciences, University of Kragujevac, Svetozara Markovića 69, 34000 Kragujevac, Serbia; nzdravkovic@gmail.com (N.Z.); saramijailovic212@gmail.com (S.M.); 4 Department of Physiology, Faculty of Medical Sciences, University of Kragujevac, Svetozara Markovića 69, 34000 Kragujevac, Serbia; vladimirziv@gmail.com; 5 Department of Pharmacology, 1st Moscow State Medical, University IM Sechenov, 8 Trubetskaya Street, Str. 2, 119991 Moscow, Russia; 6 Department of Pharmacy, Faculty of Medical Sciences, University of Kragujevac, Svetozara Markovića 69, 34000 Kragujevac, Serbia; tnikolict@gmail.com; 7 N.A. Semashko Public Health and Healthcare Department, F.F. Erismann Institute of Public Health, I.M. Sechenov First Moscow State Medical University (Sechenov University), 119991 Moscow, Russia; 8 Department of Human Pathology, 1st Moscow State Medical, University IM Sechenov, 8 Trubetskaya Street, Str. 2, 119991 Moscow, Russia

**Keywords:** blood pressure monitoring, hypertension, measuring, novel technologies in BP monitoring, telemonitoring, systolic and diastolic pressure

## Abstract

Background: The purpose of this study was to compare home and office BP in the adjustment of antihypertensive treatment. Methods: This study was an open, prospective, noninterventional, multicenter clinical trial that occurred between July 2019 and February 2020, in 34 cities in the territory of the Republic of Serbia, which monitored 1581 participants for 6 months. Depending on the used blood pressure monitoring method used, all patients were divided into control (office BP monitoring) and experimental (home BP telemonitoring) groups. We collected anamnestic data and data about systolic blood pressure (SP), in mmHg, diastolic blood pressure (DP), in mmHg, and heart rate (HR), in beats/minute, from all patients. Results: SP values were significantly different at baseline, and at the second, third, and fourth visits between the two tested groups. Home and office BP decreased significantly (*p* < 0.000) during the 6-month follow-up. We observed a statistically significant influence of the presence of diabetes mellitus and dyslipidemia on the dynamics of differences between SP monitoring values. Conclusions: Our study suggests that novel technologies in BP monitoring can be excellent alternatives for BP assessment in hypertensive patients with other cardiovascular risk factors such as diabetes and dyslipidemia.

## 1. Introduction

### 1.1. Background

Currently, hypertension (HTA) is the most significant and prevalent risk factor for cardiovascular diseases (CVD). Data from 2015 indicate that 1.13 billion people in the world suffer from HTA [1,2], and the prevalence of hypertension in Europe ranges from 9% to 20% in the adult population and from 44% up to 60% in the elderly [2]. According to 2013 data, the situation in Serbia is similar—the prevalence of prehypertension in Serbia was 33.1%, and HTA was as high as 49.3% in the adult population [2,3].

Adequate blood pressure (BP) measurement method is essential for the correct assessment of hypertension and the need for antihypertensive treatment. Home measurement and office BP monitoring only complement the conventional office BP method regarding the optimal technique for measuring BP [4,5].

Today, there is growing evidence of the benefits of home blood pressure and heart rate monitoring over standard measurement at medical examinations or office BP monitoring [6]. Home blood pressure monitoring involves BP self-measurement and monitoring by patients [7]. Systematic reviews and meta-analyses highlight the advantages of using this method because the target values of BP are easier to achieve, compliance is better compared with the classical approach to HTA treatment, and it better predicts mortality and morbidity [8,9]. Additionally, home measurement has several advantages, such as better reproducibility, better correlation to end-organ damage, and the absence of the white coat effect [10,11]. On the other hand, office BP measurements and control do not have negative aspects, such as the lack of digit preference and observer bias. 

Previous studies have reported that antihypertensive therapy based on 24 weeks of either office BP control or home measurement, instead of office control, led to less intensive drug treatment and less BP control with similar costs [12,13]. The use of home BP control is even more acceptable because modern digital BP measuring devices provide superior accuracy and reliability, even in special groups of hypertensive patients, such as obese or patients with large upper arm circumference [14,15,16].

Telemedicine is the application of technological and telecommunication achievements to transmit data over long distances to improve patients’ health [17]. Home telemonitoring involves the use of telemedicine to transmit data of vital and nonvital parameters of the patient at home, in order to monitor, interpret data, and make decisions about treatment [18,19]. Telemonitoring can be successfully used in chronic diseases, such as diabetes mellitus, lung diseases (asthma, chronic obstructive pulmonary disease), heart failure, and HTA [20,21]. It is usually applied at the primary health care level, encouraging patients to take control of their health [20].

A new approach to the treatment of HTA is home monitoring with the application of new technologies—the so-called “home telemonitoring of BP”. This new method enables BP value data transmission over long distances and their reading by a doctor, who has access to data whenever necessary. This allows a better degree of data analysis and graphical display of BP variation at home, thus achieving more accurate diagnostic and therapeutic decisions without additional hospital visits [22]. Thus far, the advantages of home measurement with the application of telemonitoring over the conventional measurement of BP in the outpatient clinic in comparison to other methods have been described [23]. In addition, the use of digital forms of communication can be considered acceptable for young people in whom the incidence of HTA is increasing [23]. 

In s systematic review, Paré et al. point out that the application of telemonitoring leads to an improvement in the control of HTA and other diseases for which this approach has been applied [24].

In the Republic of Serbia, one of the most critical problems is that a large percentage of hypertensive patients have inadequate BP control. Patients are not compliant or sufficiently motivated because the therapy is chronic, and the symptoms are often mild and nonspecific. Doctors do not have enough time to solve the problem of patients’ compliance with taking therapy. In order to adequately control (monitor) BP, it is proposed to use digital forms of communication such as modern applications on “smartphones”. A better motivation of both the doctor and the patient is expected, as well as better patient compliance, conditioned by the information offered by modern technologies.

### 1.2. Objectives

Based on all mentioned facts, we hypothesized that home BP control supported by modern technologies in patients with different comorbidities could be a better choice for achieving the target values of BP, compared with the traditionally controlled patients. Statistically significant better results in achieving the target values of blood pressure are expected in patients who measure and monitor their blood pressure more regularly and more often receive advice/reminders from their doctor via digital devices than in traditionally controlled patients.

The purpose of this study was to (1) compare home and office BP in the adjustment of antihypertensive treatment and (2) compare the differences in measuring BP values using a smartphone application, with active advice/reminders from doctors and regular entry of BP values, with the effects achieved in standard HTA treatment.

## 2. Methods

### 2.1. Trial Design

This study was an open, prospective, noninterventional, multicenter clinical trial that occurred between July 2019 and February 2020 in 34 cities in the territory of the Republic of Serbia and in 47 health care institutions at primary, secondary, and tertiary levels. 

### 2.2. Participants

The study cohort included 1581 participants of both genders who were previously diagnosed with unregulated arterial hypertension grade I/II (values of blood pressure: 140/90 mmHg–179/109 mmHg) with(out) cardiovascular complications of hypertension. The main inclusion criteria were age from 18 to 90 years, both genders, diagnosed treated or untreated arterial hypertension, and written informed consent. The main exclusion criteria were systolic blood pressure (SBP) above 180 mmHg or diastolic blood pressure (DCP) above 110 mmHg; expected difficulties in BP home monitoring; patients working at night or shift workers; body mass index above 35 kg/m^2^; atrial fibrillation and/or other significant arrhythmias; pregnancy; inability to use telemedicine systems; significant cardiovascular, cerebrovascular, or peripheral vascular event in the previous 6 months; significant sleep apnea syndrome and any condition, including alcohol or drug use, that may interfere with the completion of the study. 

### 2.3. Study Protocol

The initial pre-entry screening was carried out by 85 physicians (cardiologists, internists, and general practitioners), who enrolled and monitored 1581 participants for 6 months (minimum of 10 patients per physician). All patients were treated on an outpatient basis in one of the Centers for Hypertension in the Republic of Serbia. 

Patients who met the inclusion criteria were assigned to one of the following groups: 

Group 1 home BP telemonitoring group (experimental group): Patients whose HTA was managed based on home BP telemonitoring data. These patients had an installed application “mojpritisak.rs”. Patients would have measured their BP at least twice a week at home during the study and entered the appropriate data (SBP, DBP, and HR values) into their smartphone app. Patients in this group could track their BP measurement data, which were also presented in a form of a graph. Doctors had access to these data at their clinics and were obliged to monitor the patient’s measurements. By communication window within the app, doctors were able to send instructions via SMS/email to patients if their BP was not well regulated. In addition, patients also received generated SMS/emails with tips for cardiovascular risk reduction and lifestyle changes, based on the presence of different risk factors (diabetes, hyperlipidemia, smoking, and obesity). Moreover, smartphone apps, BP devices, and patient progress would be checked on follow-up visits. 

Group 2 usual care (control group): Patients whose HTA management was based on office BP measurements performed at regular checkups on an outpatient basis. After inclusion, physicians monitored patients from this group over 4 visits, when their BP was measured and therapy was evaluated, according to the study protocol.

Patient selection was made based on inclusion and exclusion criteria and the patient’s willingness to home monitor BP. 

As for the generated SMS/emails with tips for cardiovascular risk reduction and lifestyle changes, they contained short tips for lipid reduction, the importance of physical activity, and the danger of obesity and diabetes. Messages were sent on a monthly basis, via email or SMS. Doctors were only obliged to send messages to patients regularly when they would see poorly regulated HTA in patients’ virtual files. 

Patients who met the inclusion criteria and who had no identifiable cause for exclusion were included in the study and were followed during the period of 24 weeks, which included four visits to the doctor:

1. The first visit was the introduction of the patient to the study (baseline values of BP and HR);
2. The second visit occurred after 30 + 14 days;
3. The third visit occurred after 90 + 14 days;
4. The fourth visit occurred after 180 + 14 days.

### 2.4. Collecting of Data (Primary and Second Outcomes)

During the first visit, we obtained a medical history for all patients and performed a standard physical examination. A detailed history was collected from all patients using a questionnaire with a special emphasis on data related to cardiovascular risk factors such as hypertension, smoking, dyslipidemia, physical activity, stress, diabetes, family history, drug tolerance, salt and alcohol consumption, presence of depression and other mental disorders, social history, sleep apnea screening, level of health education and social history, BMI, waist and hip circumference, and age and sex of patients. 

### 2.5. Home and Office Blood Pressure Measurements

Before the pre-entry screening period, all patients received individual guidance about the study protocol and how to measure BP correctly. Blood pressure monitoring was applied according to the recommendations of the European Society of Hypertension [25]. That means the BP should have been measured after the general rules have been satisfied: The patient should sit or stand with the arm held at heart level; the patient should not smoke or take caffeine for 30 min before measuring BP; BP measurement should be performed after at least 5 min of rest; the appropriate cuff size should be used; the cuff bladder should cover at least 80% of the arm circumference. Patients measured their BP on two consecutive measurements twice a day and calculated the mean value. If the first two measurements differed by more than 15 mmHg, they would take additional measurements and average BP. Measurements were performed in the morning between 6 and 11 a.m., before taking medication, and in the evening between 5 and 10 p.m., before taking the evening meal. It was recommended to measure BP at least 3 days a week and preferably for 6–7 consecutive days before each clinic visit.

From all patients, we collected data about systolic blood pressure (SP), in mmHg, diastolic blood pressure (DP), in mmHg, and heart rate (HR), in beats/minute. An average of two or more measurements was taken. If the first two measurements differed by more than 15 mmHg, we took additional measurements and averaged BP or HR.

Home BP telemonitoring was performed by a validated home device (Microlife Blood Pressure Monitoring device for upper arm, BPB6-40) for measuring BP and entering all data into the mobile phone application. After 5 min of rest in the sitting position, patients performed two consecutive self-measurements of BP twice a day, in the morning between 6 and 11 a.m., before taking medication, and in the evening between 5 and 10 p.m., before taking the evening meal. Patients wrote down the BP values and time of day. The self-measured BP included the average values of all readings during all follow-ups of six months. Patients received appropriate verbal and written advice regarding blood pressure measurement. 

Office BP monitoring was performed in 0, 1, 3, and 6 months from the first visit on all patients using a validated Bp measuring device (Microlife Blood Pressure Monitoring device for upper arm, BPB6-40) in the morning (6 a.m. to 11 p.m.) and at night time (11 p.m. to 6 a.m.). Blood pressure was measured during each patient’s visit to the clinic the using BP measurement method available at the clinics.

### 2.6. Statistical Analysis

Database analysis and management were performed using SPSS Statistics software, version 22.0 (IBM Support, New York, NY, USA). All data were tested for normality and then presented using methods of descriptive statistics (measures of frequency, dispersion of variations, and measures of central tendency). The between-group differences in continuous measurements were calculated by subtracting the mean changes from baseline in the home BP group from those of the office BP group using Mann–Whitney or Wilcoxon tests for continuous variables or χ^2^ test in the case of categorical variables. The influence of categorical variables on the dynamics of BP changes and differences in both groups was tested using multivariate analysis of variance (MANOVA). The statistical significance level was set to 0.05 (5%). 

The study sample was calculated according to the assumption of a margin error of 5% and a confidence interval of 95%. Using the online Raosoft sample size calculator, a sample of 1450 respondents was calculated, which was rounded up to 1500 participants.

## 3. Results

### 3.1. Baseline Patient Characteristics

In this study, 1581 patients met the inclusion criteria and underwent randomization, 780 in the office BP (control) group and 801 in the home BP telemonitoring (experimental) group (Table 1). The baseline characteristics of the patients in the office and home BP groups were similarly distributed (gender, age, height, weight, and BMI) in relation to gender in comparison to these two groups. Additionally, we observed a statistically significant difference in the frequency of male and female patients in groups separately (Table 1). Most patients were without cardiovascular risks such as DM and smoking, and with dyslipidemia and a family history of premature CVD as risk factors (Table 1).

### 3.2. Blood Pressure Control

Table 2, Table 3 and Table 4 show the means of SP, DP, and HR in control and experimental groups during the follow-up period.

In comparison to baseline values, SP values were significantly lower after follow-up (at the fourth visit), as were values of DP and HR in all groups. Additionally, SP values were significantly different at baseline, as well as at the second, third, and fourth visits between the two tested groups (Table 2). On the other hand, DP values were significantly different at the time of the first three visits between the control and experimental group (Table 3). HR values were significantly changed between the control and experimental group between baseline and fourth measurements (Table 4). Home and office BP decreased significantly (*p* < 0.000) during the 6-month follow-up. 

**Figure 1:**
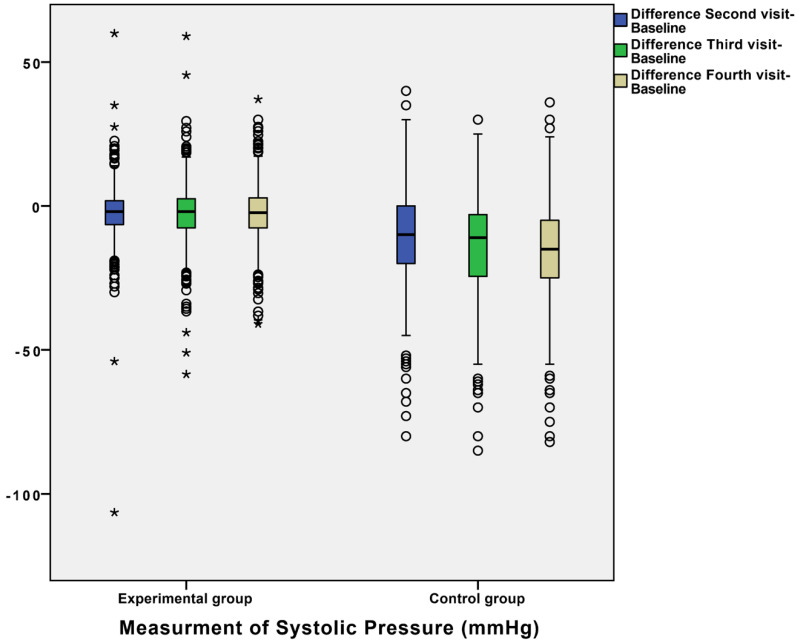
Box plot for Differences between measurements of systolic pressures (SP) in experimental and control groups during the follow-up of patients. Statistical difference was confirmed by Wilcoxon test and marked with asterisk (* = *p* < 0.05).

**Figure 2:**
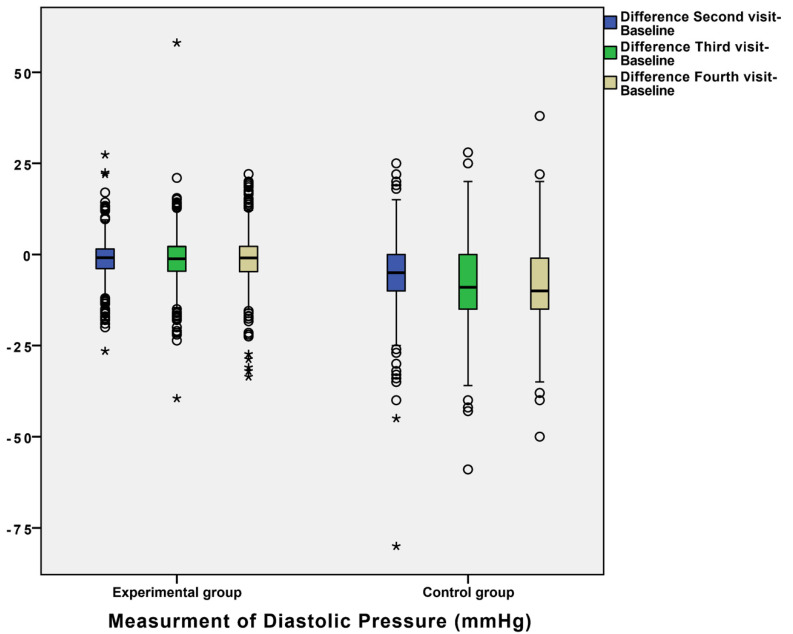
Box plot for differences between measurements of diastolic pressure (DP) in experimental and control groups during the follow-up of patients. Statistical difference was confirmed by Wilcoxon test and marked with asterisk (* = *p* < 0.05).

**Figure 3:**
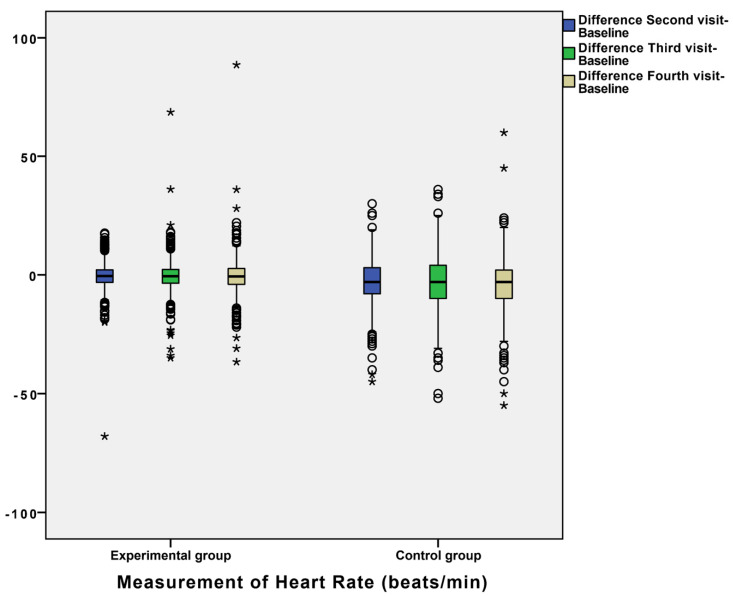
Box plot for differences between measurements of heart rate (HR) in experimental and control groups during the follow-up of patients. Statistical difference was confirmed by Wilcoxon test and marked with asterisk (* = *p* < 0.05).

In order to explain the results as precisely as possible, we presented results of the dynamics of BP in the form of a comparison of differences between measurements in the control and experimental group (Figure 1, Figure 2 and Figure 3). 

For example, the mean differences in systolic blood pressure between the second and baseline measurement were −10.712 ± 0.582 in the control group and −2.637 ± 0.341 in the experimental group (Figure 1), with a statistically significant difference between these values. Additionally, differences between the third and baseline visit and the fourth and baseline visit between the two groups were statistically significant (Figure 1). Additionally, we observed other statistically significant differences between the second, third, and fourth visits, and baseline regarding DP and HR (Figure 2 and Figure 3). For example, the mean of DP differences were −6.089 ± 0.375 in the control group and −1.293 ± 0.196 in the experimental group, which was statistically significant. The mean differences between measurements of blood pressure and heart rate (SP, DP, and HR) in the experimental and control groups during patient follow-up are shown in Figure 1, Figure 2 and Figure 3. Definitely, home measurements in patients (experimental group) with HTA showed more homogenous BP and HR values than office measurements in matched patients (Figure 1, Figure 2 and Figure 3). 

### 3.3. Influence of the Cardiovascular Risk Factors on Differences of SP and HR Values in Patients with HTA

In our study, we performed a multivariate analysis of variance (MANOVA) in the study population depending on the presence of diabetes mellitus, dyslipidemia, smoking, hereditary factors, and time. We observed a statistically significant influence of the presence of diabetes mellitus and dyslipidemia on the dynamics of differences between SP monitoring values (Table 5). 

On the other hand, a nonsignificant trend persisted in diabetes mellitus and dyslipidemia relative to DP and HR values, as well as in the influence of the presence of smoking and positive hereditary factors in relation to SP, DP, and HR values (Table 5). 

Combined analysis of variance for systolic pressure yielded a significant interaction between the presence of diabetes mellitus and time, Wilks’ lambda = 0.992, *p* = 0.011, partially eta square = 0.008. There was also a significant separate (basic) effect of time, Wilks’ lambda = 0.765, *p* = 0.000, partially eta square = 0.235, with a decrease in patient pressure obtained during the four examinations in both groups. The separate effect of diabetes mellitus was significant, *p* = 0.000, which means that this comorbidity affects systolic pressure (Table 5).

Combined analysis of variance for systolic pressure yielded a significant interaction between the presence of dyslipidemia and time, Wilks’ lambda = 0.990, *p* = 0.004, partially eta square = 0.010. There was also a significant separate (basic) effect of time, Wilks’ lambda = 0.746, *p* = 0.000, partially eta square = 0.254, with a decrease in patient pressure obtained during the four examinations in both groups. The separate effect of dyslipidemia was significant, *p* = 0.000, which means that this comorbidity affects systolic pressure (Table 5).

A combined analysis of variance for diastolic pressure did not show a significant interaction between the presence of diabetes mellitus/dyslipidemia and time, Wilks’ lambda = 0.995, *p* = 0.097, partially eta square = 0.200. A significant separate (basic) influence of time was found, Wilks’ lambda = 0.800, *p* = 0.000, partially eta square = 0.200, with a decrease in the pressure of patients obtained during the four examinations in both groups. The separate effect of diabetes mellitus was not significant, *p* = 0.472, which means that this comorbidity does not affect diastolic pressure (Table 5).

## 4. Discussion

In this open, prospective, noninterventional, multicenter clinical study, parallel groups of patients with diagnosed HTA were monitored for home or office BP measuring. The purpose was to compare home BP telemonitoring and office BP in the adjustment of antihypertensive treatment, as well as was to compare the differences in measuring BP values using a “smartphone” application with active advice/reminders from doctors and regular entry of BP values, with the effects achieved in standard HTA treatment. 

Blood pressure monitoring in the clinic has relied primarily upon using the auscultatory methods on various sphygmomanometers. This method is still the “gold standard” for blood pressure monitoring [26,27]. On the contrary, modern technologies can take an average of several measurements and eliminate the white-coat effect on BP, and future investigation must be aimed to explore new precise technologies/methods for hypertensive patients [28,29,30]. 

Today, home blood pressure monitoring is an interesting option in monitoring patients with hypertension and provides good control of BP in an environment familiar to the patient [31]. This method of measuring could be a good choice for some special populations and situations, such as patients with diabetes, noncompliant patients, patients with white coat fear, and masked form of hypertension [32,33]. By this method, during self-measuring, the patient could document BP along with the pulse rate, time, and date. Additionally, complying with home blood pressure measuring is usually very high [34].

Nevertheless, all these methods have been used to evaluate and minimize the risk for hypertension-related morbidity and mortality, and still, no clear data exist on the difference between measuring blood pressure at home using modern technologies in comparison with traditional office blood pressure monitoring.

### 4.1. Influence of Home BP Telemonitoring on BP Reduction

This study showed that home BP telemonitoring significantly reduced BP in patients with uncontrolled HTA. Several studies in the past 5 years have shown similar results [18,19,20,21,22].

In the randomized controlled trial TASMINH 2 [35], McManus et al. examined the sustainability of BP reduction in self-monitoring and self-titration of medication based on home BP telemonitoring, compared with usual care of HTA management. The group that self-monitored BP with the use of telemonitoring and self-titrated antihypertensive therapy had a significantly higher reduction in SBP after 12 months, compared with the control group (17.6 mmHg versus 12.2 mmHg). 

Affirmative results were continued throughout the TASMINH 4 study, in which three groups of patients were compared: patients who controlled BP through home BP monitoring, home BP telemonitoring, and classical management of HTA at medical examinations. After 12 months of follow-up, they showed statistically significantly lower values of mean SBP and DBP in groups with home BP monitoring with and without telemonitoring, compared with office BP measurement. The decrease was slightly higher in the group that used telemonitoring, compared with the group without it (−4.7 mmHg versus −3.5 mmHg), but there was no statistical significance. In addition, their results indicate better titration of therapy when using home BP monitoring and telemonitoring and better patient compliance, as well as the possibility of application at the level of primary health care with a reduction in the workload of physicians [36].

Furthermore, a study on the cost effectiveness of the TASMINH 4 trial economically justified self-monitoring of HTA, with or without telemonitoring, for monitoring and treatment of HTA with a reduction in cardiovascular mortality [37].

Similar to previous studies, a randomized controlled trial conducted by McKinstry et al. compared the effect of telemonitoring and standard HTA management on the reduction in BP values measured by office BP monitoring; after 6 months of follow-up, SBP was lower by 4.3 mmHg and DBP by 2.3 mmHg [18]. Although the reduction was significant, which indicates the effectiveness of telemonitoring, an economic study that followed showed that the use of telemonitoring is more expensive than standard treatment [38,39].

In a cluster-randomized study, in addition to the use of home BP telemonitoring, consultation with a pharmacist was used as an intervention. After 12 months of follow-up, it showed a difference in SBP by 9.7 mmHg and DBP by 5.1 mmHg, and a significant difference persisted for 18 months after intervention [40].

Recent meta-analysis studies [6,12,41] also support the benefits and success of telemonitoring and other supplements for the self-measurement of BP by patients. In their meta-analysis, Tucker et al. found that home BP monitoring with the application of cointerventions leads to a significant reduction in BP, which lasts for at least 12 months; the effect of reduction was in correlation with the intensity of cointerventions [6]. In our study, in addition to smartphone apps, patients received advice on healthy lifestyles and had the option to communicate with the research physicians via email or SMS, which was important for better compliance.

Adequate therapy and control of BP significantly affect the occurrence of CVD: reduction in SBP by 10 mmHg and DBP by 5 mmHg reduces the risk of cardiovascular events by ~20%, total mortality by ~10–15%, risk of stroke by ~35%, risk of a coronary event by ~20%, and heart failure by ~40% [5]. This reflects the benefit and role of telemonitoring in the treatment of HTA and cardiovascular risk in general.

### 4.2. Influence of Telemonitoring on the Improvement of Adherence Therapy

One of the problems of poor regulation of BP is poor therapeutic adherence of patients [41]—some research studies show that as many as 50–80% of patients with prescribed antihypertensive therapy have low adherence to the treatment regimen [42]. In a meta-analysis, Fletcher et al. examined the impact of home BP monitoring on therapeutic adherence and lifestyle change; they showed a statistically significant increase in adherence and a decrease in BP values after the application of HBPM [41]. In our study, patients were provided with advice to take their therapy regularly and additional notification when they entered elevated BP values. A study by Morrissey et al. identified a potential benefit of using a smartphone application for HTA monitoring that also contained a medication reminder [42]. On the other hand, in a meta-analysis study, Plumer et al. indicated that there is not enough quality evidence that interventions conducted via mobile phones affect greater therapeutic adherence and primary prevention of CVD [42].

### 4.3. Applicability of Telemonitoring at Different Ages

The obvious disadvantage of introducing new technologies such as smartphones in the control of HTA is their poor applicability among the elderly, due to poorer technological literacy [36], although they make up a large percentage of hypertensive patients. In contrast, the potential benefit can be observed in the younger adult population, who are using smartphones in an increasing manner [14]. A US survey on app users found that 31% of cell phone owners use a phone to inquire about health, of which 52% use smartphones. Therefore, telemonitoring has great potential because of the fact that, in the population of young adults (18 to 39 years), about 20% of men and 15% of women are diagnosed with HTA, and the incidence has a growing trend due to the increase in obesity and other lifestyle factors [27,28,29]. In addition, in this group, control of HTA, and therapeutic compliance, are worse than those in the population older than 40 years [30], and only 48% of young adults with HTA manage to achieve disease control within 24 months [31]. Johnson et al. revealed that younger adults without comorbidity are diagnosed with HTA significantly later than older patients. In addition, younger adults with occasionally normal BP values have a 26% slower diagnosis. Slower diagnosis in younger adults highlights the need for outpatient pressure monitoring [21]. In our study, the average age in the group with home BP telemonitoring was around 54, which indicates that even older adults were capable and motivated to use smartphone app support for BP monitoring. 

### 4.4. Acceptance of the Application of Technology by Patients and Physicians

Numerous studies have examined the impact of new technologies on patient behavior. A qualitative study examining patients’ impressions on using telemonitoring highlighted the usefulness of the application in terms of communication with medical staff and empowerment of patients to self-monitor BP but raised concerns about increased health anxiety and technology unsustainability over time [24]. Conversely, some randomized, controlled studies have shown a small percentage of anxiety occurrences [16,18,36].

As regards issues related to doctors, the greatest concern was fear of potentially increased workload [24], which has not been shown in practice [16]. In a qualitative study, doctors highly valued fast access to data on a monthly basis, better visibility of graphical representations compared to diaries written on paper, as well as better and faster communication with patients [36].

In our study, there were no significant objections from patients and physicians. All doctors received instructions and training in running the application on the patient’s phone, which they accomplished without major difficulties. The simplicity of application use was praised by doctors and patients, as well as the option of additional communication with the patient.

### 4.5. Advantages of the Study

Based on the results of our cohort study, it seems that home monitoring should be used in conjunction with office monitoring as a complementary method of BP assessment. Home BP monitoring may have practical advantages in being used on a regular basis in the clinical setting. Home blood pressure monitoring may be easier to implement and, with increasing telehealth and computing capabilities, may be incorporated, for example, into the care of hypertensive dialysis patients, as one of the seriously ill populations. However, whether such an approach results in reducing hypertension-related adverse clinical outcomes remains to be seen in future clinical studies.

To the best of our knowledge, this is the first study of the implementation of new technologies for HTA management and CVD risk factors reduction in the Republic of Serbia.

The study included patients from 34 cities in all regions of the country, which enables an overview of the success of HTA control, as well as other important data (such as distribution of risk factors for CVDs) at the state level. Of the doctors who participated in the study, 85 belonged to all levels of health care (primary, secondary, and tertiary) at which the control and treatment of patients with HTA were carried out. They are also doctors of various specialties—general practitioners, specialists in internal medicine, cardiology, and endocrinology, which makes our analysis more comprehensive. Furthermore, the recent COVID-19 pandemic showed the benefits of using telemedicine in different health areas [43,44,45] which highlights the benefits of this approach in HTA management. 

### 4.6. Study Limitations

In this study, the examined group used BP measuring devices with data storage, but the measured values themselves were entered into the application on the phone “manually” by the patients. Similar studies have used BP devices that automatically transmit measured values to a mobile phone app via a Bluetooth device. This way of connecting the device and the application reduces the possibility of errors in data collection, and the measurement process itself is somewhat simpler. Nevertheless, our results indicate good compliance in terms of application use. The problem of the relevance of entered values was overcome by the possibility of doctor–patient communication via the app. In addition, regular control visits secured an objective overview of the regularity of measurements, therapy intake, and the success of BP regulation. Moreover, generic SMS/email messages with tips for risk factor reduction were sent monthly and not more often due to technical limitations of the system we used.

Another disadvantage of the study is that patients were followed for 6 months, which is enough to assess the success of the application of new technologies but not to assess the sustainability of this method of monitoring BP in practice. Further research in this direction is planned for the future.

## 5. Conclusions

This study successfully showed that home BP telemonitoring has a significant impact on lowering SBP and DBP in patients with unregulated HTA. It also showed positive responses and acceptance of telemonitoring of BP by patients. This may represent a foundation for further research on the implementation of this approach in primary practice in Serbia. 

## Figures and Tables

**Table 1:** Demographic characteristics of study population. Results are presented as mean ± standard means of errors (X ± SEM) in control and experimental groups. Statistical difference was confirmed by chi-squared and Mann–Whitney U tests.

Parameter	Control Group *n* = 780	Experimental Group *n* = 801	Statistical Significance in Groups (C/E)		Statistical Significance between Groups (C vs. E)
			C	E	
Gender (M/F)	M 325 (44.2%) F 410 (55.8%)	M 368 (64.4%) F 203 (35.6%)	χ^2^ = 9.830 *p* = 0.002	χ^2^ = 47.680 *p* = 0.000	χ^2^ = 51.807 *p* = 0.000, fi = 0.201
Age (years)	60.63 ± 0.444 M 58.92 ± 0.708 F 61.84 ± 0.592	54.13 ± 0.534 M 52.6 ± 0.679 F 56.87 ± 0.833	*p* = 0.004 Mann–Whitney	*p* = 0.000 Mann–Whitney	*p* = 0.000 Mann–Whitney
Height (cm)	171.18 ± 0.341 M 178.35 ± 0.408 F 165.45 ± 0.332	173.19 ± 0.374 M 178.07 ± 0.463 F 165.73 ± 0.415	*p* = 0.000 Mann–Whitney	*p* = 0.000 Mann–Whitney	*p* = 0.000 Mann–Whitney
Weight (cm)	81.28 ± 0.530 M 89.50 ± 0.762 F 74.66 ± 0.598	83.74 ± 0.615 M 89.62 ± 0.807 F 73.42 ± 0.914	*p* = 0.000 Mann–Whitney	*p* = 0.000 Mann–Whitney	*p* = 0.004 Mann–Whitney
BMI (cm/m^2^)	27.66 ± 0.149 M 28.14 ± 0.223 F 27.27 ± 0.211	27.74 ± 0.154 M 28.15 ± 0.194 F 26.65 ± 0.316	*p* = 0.006 Mann–Whitney	*p* = 0.000 Mann–Whitney	*p* = 0.697 Mann–Whitney
DM (yes/no)	242 (31.6%) 523 (68.4%)	132 (16.5%) 668 (83.5%)	χ^2^ = 103.217 *p* = 0.000	χ^2^ = 359.120 *p* = 0.000	χ^2^ = 48.419 *p* = 0.000, fi = 0.177
Dyslipidemia (yes/no)	507 (66.3%) 258 (33.7%)	387 (48.4%) 413 (51.6%)	χ^2^ = 81.047 *p* = 0.000	χ^2^ = 0.845 *p* = 0.358	χ^2^ = 50.427 *p* = 0.000, fi = 0.181
Smoking (yes/no)	260 (34.0%) 504 (66.0%)	272 (34.0%) 528 (66.0%)	χ^2^ = 77.927 *p* = 0.000	χ^2^ = 81.920 *p* = 0.000	χ^2^ = 0.000 *p* = 1.000, fi = 0.00
Family history of premature CVD (yes/no)	533 (69.7%) 232 (30.3%)	560 (70.0%) 240(30.0%)	χ^2^ = 118.433 *p* = 0.000	χ^2^ = 128.000 *p* = 0.000	χ^2^ = 0.007 *p* = 0.932, fi = −0.004

**Table 2:** Values of systolic blood pressure in the study population. Results are presented as mean ± standard means of errors (X ± SEM) in control and experimental groups. Statistical difference was confirmed by Mann–Whitney U test. An eta-squared (η^2^) value reflects the strength or magnitude related to a main or interaction effect.

Systolic Pressure (mmHg)	Control Group	Experimental Group	Statistical Significance	η^2^
Baseline	151.03 ± 0.617	133.80 ± 0.406	*p* = 0.000	0.775
Second visit	140.18 ± 0.486	131.10 ± 0.397	*p* = 0.000	0.548
Third visit	136.81 ± 0.440	131.03 ± 0.379	*p* = 0.000	0.381
Fourth visit	134.69 ± 0.427	130.92 ± 0.390	*p* = 0.000	0.255

**Table 3:** Values of diastolic blood pressure in the study population. Results are presented as mean ± standard means of errors (X ± SEM) in control and experimental groups. Statistical difference was confirmed by Mann–Whitney U test. An eta-squared (η^2^) value reflects the strength or magnitude related to a main or interaction effect.

Diastolic Pressure (mmHg)	Control Group	Experimental Group	Statistical Significance	η^2^
Baseline	90.15 ± 0.359	81.79 ± 0.283	*p* = 0.000	0.622
Second visit	84.04 ± 0.332	80.40 ± 0.280	*p* = 0.000	0.336
Third visit	81.48 ± 0.296	80.40 ± 0.285	*p* = 0.002	0.115
Fourth visit	80.38 ± 0.285	80.38 ± 276	*p* = 0.812	0.008

**Table 4:** Values of heart rate in the study population. Results are presented as mean ± standard means of errors (X ± SEM) in control and experimental groups. Statistical difference was confirmed by Mann–Whitney U test. An eta-squared (η^2^) value reflects the strength or magnitude related to a main or interaction effect.

Heart Rate (Beat/min)	Control Group	Experimental Group	Statistical Significance	η^2^
Baseline	76.72 ± 0.415	74.03 ± 0.309	*p* = 0.000	0.155
Second visit	73.36 ± 0.318	73.31 ± 0.320	*p* = 0.762	0.011
Third visit	73.46 ± 0.342	73.39 ± 0.334	*p* = 0.929	0.003
Fourth visit	72.423 ± 0.345	73.33 ± 0.339	*p* = 0.009	0.097

**Table 5:** Multivariate analysis of variance (MANOVA) in study population depending on presence of diabetes mellitus, dyslipidemia, smoking, hereditary factors, and time. An eta-squared (η^2^) value reflects the strength or magnitude related to a main or interaction effect (0.01 = small, 0.06 = medium, 0.13 = large).

Parameters		Value	F	df	Error df	*p*	η^2^
DM	SP	0.992	3.735	3.00	1394.0	0.011	0.008
	DP	0.995	2.107	3.00	1394.0	0.970	0.005
	HR	0.997	1.288	3.00	1385.0	0.277	0.003
Dyslipidemia	SP	0.990	4.535	3.00	1393.0	0.004	0.010
	DP	0.996	1.707	3.00	1393.0	0.164	0.004
	HR	1.000	0.134	3.00	1384.0	0.940	0.000
Smoking	SP	0.995	1.128	3.000	725.000	0.337	0.005
	DP	0.992	0.789	3.000	724.000	0.238	0.004
	HR	0.996	0.145	3.000	727.000	0.605	0.002
Positive hereditary factors	SP	0.991	2.179	3.000	725.000	0.089	0.009
	DP	0.990	1.679	3.000	724.000	0.075	0.011
	HR	0.998	0.156	3.000	727.000	0.451	0.001

## Data Availability

All data associated with the paper are available on request from the corresponding author. The study was written and conducted according to all CONSORT criteria.
